# Rare Earth Element Variability in Italian Extra Virgin Olive Oils from Abruzzo Region

**DOI:** 10.3390/foods13010141

**Published:** 2023-12-30

**Authors:** Alessandro Chiaudani, Federica Flamminii, Ada Consalvo, Mirella Bellocci, Alberto Pizzi, Chiara Passamonti, Angelo Cichelli

**Affiliations:** 1 Department of Innovative Technologies in Medicine and Dentistry, University “G. d’Annunzio” of Chieti-Pescara, 66100 Chieti, Italy; alessandro.chiaudani@unich.it (A.C.); angelo.cichelli@unich.it (A.C.); 2 Center for Advanced Studies and Technology (CAST), “G. d’Annunzio” University of Chieti-Pescara, 66100 Chieti, Italy; ada.consalvo@libero.it; 3 Istituto Zooprofilattico Sperimentale dell’Abruzzo e del Molise “G. Caporale”, Campo Boario, 64100 Teramo, Italy; m.bellocci@izs.it; 4 Department of Engineering and Geology, University “G. d’Annunzio” of Chieti-Pescara, 66100 Chieti, Italy; alberto.pizzi@unich.it; 5 Department of Philosophical, Pedagogical and Economic-Quantitative Sciences, University “G. d’Annunzio” of Chieti-Pescara, 65127 Pescara, Italy; chiara.passamonti@unich.it

**Keywords:** extra virgin olive oil, ICP-MS, rare earth element, lanthanides, chemometric techniques

## Abstract

Extra virgin olive oil is a food product from the Mediterranean area that is particularly and continuously experiencing to increasing instances of fraudulent geographical labeling. Therefore, origin protection must be improved, mainly based on its intrinsic chemical composition. This study aimed to perform a preliminary chemical characterization of Abruzzo extra virgin olive oils (EVOOs) using rare earth elements (REEs). REEs were evaluated in EVOO samples of different varieties produced in different geographical origins within the Abruzzo region (Italy) in three harvest years using ICP-MS chemometric techniques. Principal component, discriminant, and hierarchical cluster analyses were conducted to verify the influence of the variety, origin, and vintage of the REE composition. The results of a three-year study showed a uniform REE pattern and a strong correlation in most EVOOs, in particular for Y, La, Ce, and Nd. However, europium and erbium were also found in some oil samples. Compared with cultivar and origin, only the harvest year slightly influenced the REE composition, highlighting the interactions of the olive system with the climate and soil chemistry that could affect the multielement composition of EVOOs.

## 1. Introduction

Extra virgin olive oil (EVOO) is an important agricultural product. Its consumption is common in everyday cooking [1,2]. It represents the main fat source of the “Mediterranean diet”, providing a special aroma and taste due to the contribution of its phytochemical compounds [3]. Its high contents of monounsaturated fatty acid and antioxidant compounds has beneficial health effects [4,5,6] in preventing coronary diseases, cancer types, diabetes, and autoimmune illness [7,8,9]. Worldwide, olive oil production was estimated as almost 3 million tons for the period from 2021 to 2022, with about 2 million tons produced in Europe (Spain, 1400 t; Italy, 329 t; Greece, 232 t; and Portugal, 206 t) and about 1 million tons in non-European countries (Tunisia, 240 t; Turkey, 235 t; Morocco, 200 t; Algeria, 91 t; Egypt, 20 t; and Argentina, 3 t) [10].

The Abruzzo region produces the fifth largest EVOO volume in Italy, at about 144,000 tons in 2021 [11]. Owing to local varieties and the specific pedoclimatic conditions in the Abruzzo region, it is possible to cultivate olive trees over the entire territory, starting from the sea to the foothills of Majella and the Gran Sasso mountains located 600–700 m above sea level [12]. Fifty percent of the annual regional EVOO production is concentrated in the Chieti district, and the Pescara province produces thirty percent. The rest of the production is distributed in Teramo and L’Aquila districts, with 16 and 4%, respectively. The EVOO produced in the country is appreciated all over the world as a very-high-quality product [13], and, for this reason, tracing the origin of EVOO to control product authenticity or adulteration is fundamental [14,15,16,17,18]. In fact, the adulteration of EVOO with other vegetable oils is relatively easy to recognize. Conversely, it is difficult to check extra virgin oils produced from foreign countries or those using imported olives and sold as local products, because the overall composition of the final product can be almost totally similar to that of local ones [19].

Here, the focus is not only on food safety and quality control but also on the assessment of the authenticity of the declared geographical origin. Indeed, frauds can have a significant impact on customer health, confidence in the product, and, consequently, e final consumption behavior, causing significant economic losses [20,21]. For these reasons, EC Regulation 1151/2012 provides rules on marketing standards for olive oil and states the mandatory nature of origin labeling [22]. It introduces discrimination based on geographical indicators, highlighting that the characteristics of the product are also related to the geographical origin [23]. The Protected Designation of Origin (PDO) label on olive oil has become a motivating choice criterion for customers, since olive oil quality and flavor are linked to the origin of the olives, and they are associated with specific production practices [24,25].

Even though a method for certifying EVOOs’ geographical origin has not yet been established in the scientific literature, the discrimination of olive oil production on a geographic basis, also called geographical authentication, is becoming an important trend in scientific research, with different analytical chemistry approaches being applied, including elemental/isotopic, nuclear magnetic resonance (NMR), mass spectroscopy (MS), and energy-dispersive X-ray fluorescence (XRF), as well as organic analytical methods and organoleptic evaluation [22,26,27,28]. The organic analytical methods are based on the determination of fatty acids and triacylglycerols [29], but minor metabolites such as phenols [30], aliphatic and terpene alcohols, sesquiterpene hydrocarbons [31], sterols [32,33], and pigments are also considered. The isotopic profile analysis method is mostly based on the detection of stable isotope (H, C, and O) ratios, since the isotopic fractionation is correlated with geographical and climatic parameters [33,34]. The isotopic composition of strontium (Sr) is also useful, since it reflects local soil and geology [35,36,37],and, in particular, the geogenic soil formation that overlies the geological substrate [38,39].

Alternatively, authentication can be realized by evaluating the mineral composition of olive oils, which gives information regarding the biological demand of the plant, the bioavailability and mobility of mineral compounds from the soil, and the influence of agronomic practices such as the use of fertilizers and pesticides. Since olive trees of the same cultivar, characterized by the same genetics but planted in different countries, produce different oils, the analysis of their elemental profiles reflects the effect of their interaction with pedoclimatic conditions and agricultural practices [40,41,42]. For this reason, to achieve precise authentication, it is necessary to identify elements acting as natural markers that are not influenced by secondary sources such as farming practices and anthropic sources and are suitable for the determination of the origin of EVOO [26,43].

Trace elements concentration in the Earth’s crust do not exceed 1 g/kg, while, in olive oil, their concentrations, except for Ca, do not exceed a few hundred micrograms per kilogram (µg/kg) [27]. The concentrations in olive oil may differ according to several parameters such as olive cultivar [44], irrigation of olive trees [45], use of fertilizers [46], fluctuation in annual climatic parameters [47], and the positive fractionation that occurs during the active absorption from the soil and the translocation to the fruits [19]. For these reasons, no consensus has been reached with regard to a direct and clear correlation between their concentration in the soil and in the related olive oils [19,47,48,49].

Ultra-trace elements, also called rare earth elements (REEs), show wide soil concentration variability around the world, ranging from 0.1 to 700 mg kg^−1^ [50,51]. Conversely, several authors have found REE concentrations in olive oils as ranging from 0.002 to 7 ng g^−1^ [52]. Ultra-trace elements are not essential for the growth and development of olive tree and are passively absorbed without active fractionation. Therefore, the REE composition can provide a representative fingerprint of the olive oil since REEs more proportionally reflect their less-abundant concentrations in soils. Conversely, fractionation from the original distribution in soil occurs when a certain element is assumed in a preferential way because it is a nutrient, or it is excluded from absorption because it is toxic [19,26,36,41,53,54,55,56,57].

For these reasons, REEs are suitable for discriminating foodstuffs on a geographic basis, acting as geochemical markers as well [58], while major, minor, and trace elements are useful in authentication schemes based on varietal or technological discrimination [19,26]. Joebstl and coauthors used REEs to identify the geographical origin of pumpkin seed oil [59] and, lately, some studies deepened the understanding of the relationship among the production chain and EVOO characteristics [19,57]. Barbera et al. confirmed that the relationship between the soil and olive fruits depends exclusively on the soil REE composition [56] and identified an excellent marker to identify the geographical fingerprint of EVOOs [57].

REEs include ultra-trace elements (yttrium) and lanthanides with an atomic number ranging from 57 and 71, such as La (lanthanum), Ce (cerium), Pr (praseodymium), Nd (neodymium), Pm (promethium), Sm (samarium), Eu (europium), Gd (gadolinium), Tb (terbium), Dy (dysprosium), Ho (holmium), Er (erbium), Tm (thulium), Yb (ytterbium), and lutetium (Lu). Ordering elements by increasing atomic number, the REEs from La to Gd are considered light rare earth elements (LREEs), with more basic behavior and higher solubility, whereas the REEs from Tb to Lu are considered heavy rare earth elements (HREEs), with more acid behavior and lower solubility [51,60,61]. Additionally, middle rare earth elements (MREEs), which overlap the two groups, include Sm (samarium) and Eu (europium). Typically, in soils, the LREE concentrations are generally greater than the HREE concentrations [51].

Based on the previous assumptions, the aim of this study was to evaluate, for the first time, the REE contents of EVOOs from the Abruzzo region, mainly from Chieti and Pescara provinces, which produce 80% of the total EVOO in the region. In particular, 29 EVOOs produced during three harvest years (2019, 2020, and 2021) were fingerprinted with inductively coupled plasma mass spectrometry (ICP-MS). The dataset was analyzed using chemometric tools, in particular, using principal component analysis (PCA), linear discriminant analysis (LDA), and hierarchical clustering analysis (HCA), with the aim of discriminating between olive oil groups.

## 2. Materials and Methods

### 2.1. Area Sampled, Local Geology, and Sample Collection

**Figure 1:**
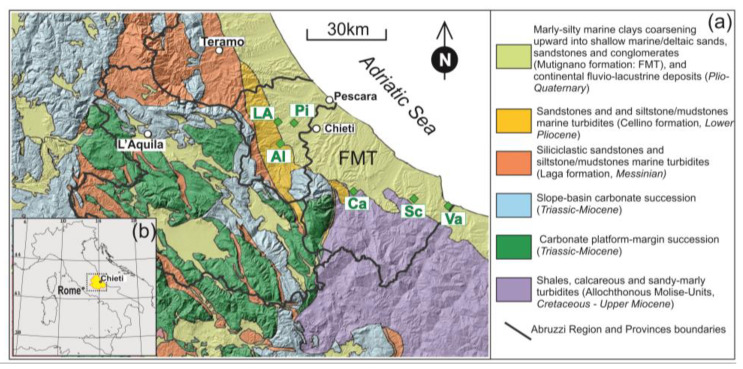
(a) Simplified geological map showing the different lithologies characterizing the Abruzzo region (modified from a geological map of Italy on a 1:50,000 scale, sheets: 361; www.isprambiente.gov.it/Media/carg/note_illustrative/361_Chieti.pdf, accessed on 09 October 2023), processed using Q-GIS tools and overlaid on a 3D topographic digital elevation model of Italy with a 10 m cell size [62]. Green diamonds indicate the localization of the analyzed olive orchards: LA, Loreto Aprutino; Pi, Pianella; Al, Alanno; Ca, Casoli; Sc, Scerni; Va, Vasto. (b) Location of Abruzzo region in Italy.

Olive harvest and EVOO extraction were conducted during the 2019, 2020, and 2021 harvesting years. The locations of the olive orchards where the drupes were collected are reported in Figure 1, which shows a simplified geological map of the Abruzzo region and its position in the coastal Adriatic region (i.e., the outer (eastern) portion of the Apennines orogenic belt (geological map of Italy: 1:50,000 scale, 361 sheets). In this region, carbonate rocks in the mountain range are exposed and sandstones alternate with marls in the adjacent low-lying mountain-foot domains. The soils of the Adriatic coastal region have developed above a common sedimentary substrate made by clayey–sandy–conglomerate shallow marine deposits of the Mutignano Formation (Late Pliocene–Lower Pleistocene). Locally, the soil is featured by silty–clayey eluvial–colluvial, alluvial, and slope Quaternary continental deposits. All the investigated olive orchards, therefore, were located on soils that had pedogenized the areas of the Mutignano Formation (FMT), with the exception of the Alanno site, which, however, developed on lithologies very similar to those of the FMT.

Olives were mechanically harvested in October from nonexperimental orchards, from 20 plants for each cultivar. Olive fruits were at a medium level of ripening, which is specific for each variety and defined by each producer. EVOOs were extracted within 12–24 h of harvest in an olive mill located in the same municipality as the olive orchard, with industrial, three-phase, continuous extraction systems. Oils were collected during the extraction, packaged in dark 750 mL glass bottles, and stored in dark conditions at about 18 °C until analysis. Oil samples were coded with alphanumeric codes based on the harvest year (Table 1); “A”, “B”, and “C” corresponded to 2019, 2020, and 2021, respectively. The trees and industrial olive mills were the same during the three years of the research. Information about the geographical location of the orchards, sample coding, and cultivars are presented in Table 1.

### 2.2. Olive Oil Preparation

Prior to inductively coupled plasma mass spectrometry (ICP-MS) analysis, the organic matter contained in the olive oils was destroyed via mineralization. The EVOO mineralization was carried out based on the EN 13805:2014 protocol adopted by the Istituto Zooprofilattico Sperimentale dell’Abruzzo e del Molise “G. Caporale” (Teramo, Italy). Briefly, a microwave digestion system, Milestone ultraWAVE ECR (Sorisole, Italy), equipped with temperature and pressure control was used to digest samples. About 300 mg of each EVOO sample was weighed directly into disposable glass vessels. The vessels were then filled with 4 mL of HNO_3_ (60%) for trace analysis (Merck KGaA, Darmstadt, Germany) and prepared for digestion. Each digestion cycle was programmed according to the protocol: step 1—ramp to temperature 230 °C and pressure 150 bar in 25 min, at 1500 W power; step 2—the same conditions maintained for 10 min; step 3—cooling cycle. Each sample resulting from acid digestion was then diluted to 15 mL with high-purity water (18.2 MΩ cm^−1^ resistivity) obtained from an ELGA LabWater PURELAB Option-Q water purification system (High Wycombe, UK).

### 2.3. ICP-MS Analysis

Digested samples (2 mL) were diluted to 5 mL with ultra-pure water (18 MΩ cm^−1^) and subjected to analysis via ICP-MS for major (Na, Mg, K), trace (Ca, Mn, Fe, Zn, Rb, Sr, Ba), ultra-trace (Al, Ga, V, Cr, Pb), and rare earth elements.

The instrument used was an Agilent 7900 ICP-MS (Agilent Technologies, Tokyo, Japan), which was used in the Laboratory of Newborn Screening, Proteomics and Endocrinology of CAST, University of Chieti. Detailed operating conditions and instrumental parameters are given in Table S1. The 4th-generation Octopole Reaction System (ORS) was used to measure, at the same time, some elements (Na, Mg, K, Ca, Fe, Zn, Rb, Al, Ga, Cr, Pb) in helium (He) mode to reduce spectral interference and noise effects; other elements (V, Mn, Sr, Ba, and REE) were measured in no-gas mode due to the lack of interference [63].

The optimization of ICP-MS was carried out to obtain the maximum signal intensities for 7Li, 89Y, 140Ce, and 205Tl using a 1 μg L^−1^ tuning solution containing Li, Y, Co, Ce, Mg, and Tl (Agilent Technologies, Palo Alto, CA, USA), while keeping the formation of oxides 140 CeO^+^/140Ce^+^ and doubly charged species Ce^2+^/Ce^+^ ratios below 1% and 2%, respectively. The sample-introduction system was washed between analyses with 2% HNO^3^. Two multielement mixtures at 10 μg mL^−1^ were used in acid solution: (A) Ag, Ba, Be, Cd, Co, Cr, Cu, Fe, Mn, Ni, Pb, Rb, Se, Sr, Tl, U, V, and Zn in 5% HNO^3^; (B) Ce, Dy, Er, Eu, Ga, Gd, Ho, In, La, Lu, Nb, Nd, Pr, Sm, Th, Tb, Tm, Y, and Yb in 5% HNO^3^. These mixtures were employed to prepare diluted calibration solutions daily, and three calibration curves were prepared using these multielement mixtures. An internal standard correction was performed via the online addition of an internal standard solution of Rh (20 μg L^−1^) in a T piece. Ultra-pure water (18 MΩ cm^−1^) was obtained from a Milli-Q system (Millipore, Bedford, MA, USA). Nitric acid (69% *v*/*v*) and an internal standard solution of Rh were bought from Merk (Darmstadt, Germany) and were ultrapure-grade. Duplicate analysis was performed for each sample. The full data were recorded with Agilent MassHunter Data Acquisition software (version 4.2) and processed with Agilent MassHunter Data Analysis software (version 4.2).

The limit of detection (LOD) and limit of quantification (LOQ) were calculated for the REEs by analyzing ten experimental blanks. The LOD and LOQ were initially calculated as signals by employing Equations (1) and (2), respectively:

yLOD = yb + 2tsb(1)

yLOD = yb + 10sb(2)

where t is the constant from a one-sided Student’s *t*-test at the 95% confidence level for n − 1 degrees of freedom, yb is the average blank signal, and sb is the corresponding standard deviation. The corresponding LOD and LOQ concentration values (Table S2) were obtained by using an appropriate calibration curve satisfying the following relationship: 0.5x_1_ < LOD < x_1_, where x_1_ is the concentration of the first calibration level [64]. Data below the LOD and LOQ values were excluded from the analysis.

### 2.4. Data Analysis

Empirical analyses were conducted to study the characteristics of the REEs according to some variables of interest, and the main features of the observed data were summarized by performing an overall descriptive analysis. The differences between mean REE concentrations related to year, variety, and origin were evaluated via analysis of variance (ANOVA) and Kruskal–Wallis tests. Where the ANOVA test assumptions were not satisfied (normality and homoscedasticity of residues), the Kruskal–Wallis nonparametric test was used. Significant differences were established at the level of *p* = 0.05.

In the second step, commonly used foodomics chemometric techniques such as PCA and HCA were used as exploratory methods, without any a priori knowledge of groups present in the population [22]. To reduce collinearity among data, PCA was applied to REE concentrations, and, based on the first two principal components, the LDA, an a priori knowledge of group membership technique, was performed [65]. Finally, the HCA method, revealing groups of similarity (clusters), was conducted using Euclidean distances and Ward’s linkage methods. The graphical output of the analysis was a heatmap, a tree-like plot, where both rows and columns were clustered [66,67].

The statistical analysis was performed with XLSTAT using the Addinsoft program and ClustVis, a web tool freely available at http://biit.cs.ut.ee/clustvis/ [68] (accessed on 1 August 2023).

## 3. Results and Discussion

### 3.1. Elemental Profile of Olive Oils

**Figure 2:**
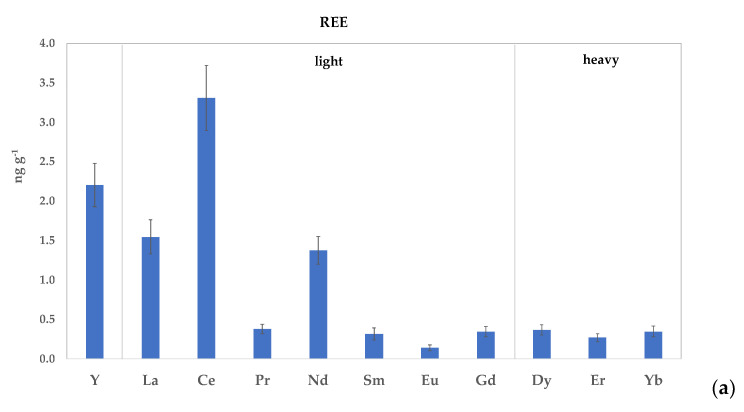

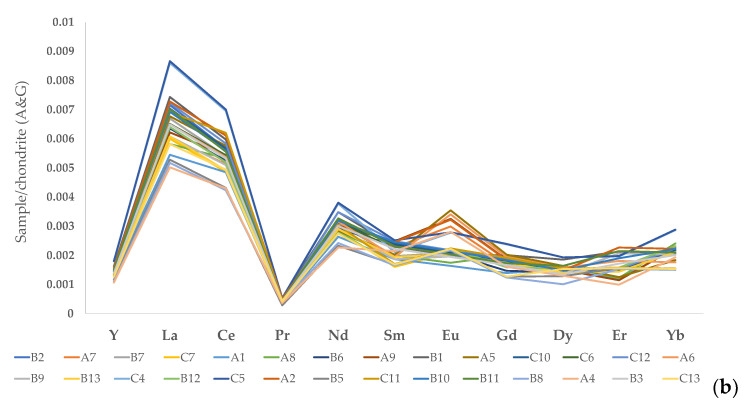
Mean REE concentrations in oils: light La, Ce, Pr, Nd, Sm, Eu, Gd; heavy: Y, Dy, Er, Yb (**a**); REE concentration patterns normalized to chondrite values (**b**).

The mean REE concentrations detected in the EVOOs are shown in Figure 2a, while the mean REE contents (≥LOQ) of each olive oil sample during the 2019–2021 period from different geographical origins are reported in Table 2. Despite the limited data on REE contents available in the literature, the concentration range that we detected ranged between 0.09 and 4.22 ng g^−1^, in accordance with the results of different authors, which were well summarized in a recent study [69], who reported ranges varying between 0.002 and 7 ng g^−1^ in olive oils from costal region. Our values also agree with those described by other authors in other areas [2,70,71,72]. In descending order, the most abundant REEs were Ce, Y, La, and Nd, which had mean values of 3.27, 2.18, 1.53, and 1.36 ng g^−1^, respectively. The levels of the remaining elements, such as Pr, Sm, Gd, Dy, Er, Yb, and Eu ranged from 0.38 to 0.13 ng g^−1^. Table S3 provides a detailed list of the REE concentrations found in the literature. To better study and visualize the REE patterns, chondrite-normalized values were used [73], which provide a reference for the normalization of rare earth elements, since they are assumed to reflect the original composition of the Earth’s crust [74,75]. Except for Eu and Er, which presented slight positive and negative anomalies, respectively, all the oil samples showed very similar chondrite normalized patterns, confirming the above-mentioned hypothesis regarding the limited fractionation that occurs when passing from soil to fruits and to the final EVO [19]. It is evident that the homogeneous lithological and geomorphological context (Figure 1) strongly affected the distribution patterns of the REEs in soils and, therefore, the general uniform REE pattern observed in the EVOOs. Slight content variations, moreover, could reflect the local influence of bedrock-weathering and soil-leaching processes.

The behavior of REE distributions strictly followed the Oddo–Harkins rule, which states how elements with an even atomic numbers are more abundant than elements with immediately adjacent atomic numbers [76,77]. The REE concentration patterns of the EVOOs (Figure 2b) therefore proportionally reflect the distribution of REEs in soils, pointing out that different vintages, varieties, and locations seem did not affect the patterns. This aspect represents one of the prerequisites for a reliable chemical marker of geographical origin, and so it would be useful to further investigate the potential of REEs in this sense.

To evaluate the strength of the relationships among the REEs, Pearson correlation coefficients were calculated and are reported in a correlation matrix (Table 3). A strong correlation (*p* < 0.001) was found among Y, La, Ce, Pr, Nd, Gd, and Dy; five of them were grouped in the light fraction (LREEs). Conversely, the weakly correlated REEs were Eu–Er (−0.04) and Eu–Yb (0.03). It was evident that similar chondrite patterns corresponded to stronger concentration correlations.

### 3.2. ANOVA and Kruskal–Wallis Test

The one-way ANOVA was conducted to evaluate the differences between mean REE concentrations related to the three vintages (2019, 2020, and 2021). The parametric test assumptions were verified for all REEs, except for Eu (failed both normality and Levene’s test) and Er (failed normality test). Ten out of eleven REEs did not exhibit any significant differences (*p* > 0.05) since there was no variation in the mean REE concentrations among the vintages. Eu exhibited significant differences between the 2019 and 2020 vintages (according on Tukey’s HSD test).

The nonparametric Kruskal–Wallis test was conducted to evaluate the differences between median REE concentrations related to the 12 varieties or cultivars (CVs) and 6 origins. No variation in the REE concentrations was found regardless of origin and the (*p* > 0.05), confirming the homogenous pattern distribution that we previously observed.

### 3.3. PCA

According to the values reported in Table 3, to reduce the collinearity among the data, principal component analysis (PCA) of the REE concentrations was conducted, and the results are shown in Table 4. The first two principal components (F1 and F2) explained 75.77% of the total variance (specifically, F1 explained 65.37% and F2 explained 10.4%). The main variables that influenced the first component were the concentrations of Y, La, Ce, and Nd (F1 eigenvectors > 0.35), while the variables Eu and Er mainly affected the second component, with eigenvector values of 0.76 and −0.54, respectively.

According to the loading plot (Figure S1), the vector lengths of Y, La, Ce, and Nd represented the largest contribution of the variance in the first component. They were located close to each other, with a small angle in between; out toward the same periphery, they covariation was strongly positive (very similar patterns) and proportional to the degree distance from the PC origin. On the other side, europium (Eu), especially, and erbium (Er), mainly contributed to the second component. The large angle between Eu and Er highlighted the weak correlation. Sm and Yb, with the shortest vector and being closest to the origin, presented the lowest absolute variances, and might be better explained by other factors [57].

The first component represented elements with strong associations, reflecting similar behavior or bioavailability in the soil, belonging to the same geochemical groups on the periodic table, being able to form clusters [47,49,78]. Consequently, according to Goldshmidt’s geochemical classification, La, Ce, and Nd (LREEs), which have more basic behavior and higher solubility, were grouped together [47]. In addition to this grouping of elements, yttrium (HREE), which also characterizes the elemental profile of olive oils, was shaped by the geochemical processes in the study territory.

**Figure 3:**
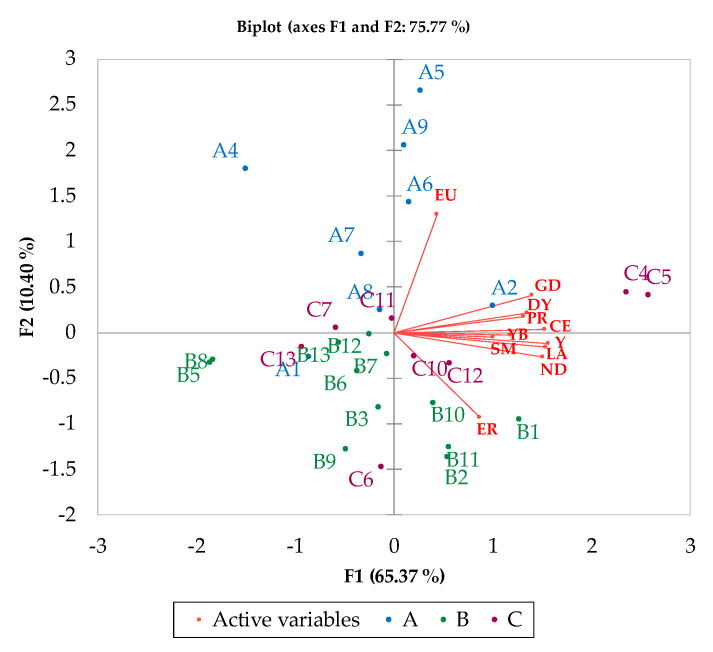
Biplot of oil samples and REEs.

A PCA biplot (Figure 3) highlights that samples C4 and C5 presented the highest concentrations of REEs and were the most positively correlated with F1. Conversely, EVOOs A4, B5, and B8 presented the lowest concentrations of REEs and were negatively correlated with F1. With respect to F2, the A samples, belonging to the 2019 vintage, specifically A4, A9, and A5, presented a highly positive correlation with europium and a negative one with erbium. Finally, the B and C samples, representing the 2020 and 2021 vintages, respectively, were mostly grouped together.

Based on the PCA assumptions, a preliminary comparison analysis was performed including the REE data of three different Italian monocultivar oils: Pisciottana (Calabria region, D1), Frantoio (Piemonte region, D2), and Taggiasca (Liguria region, D3), as well as the data from one EU/extra EU EVOO blend (D4). The PCA score plot (Figure S2) shows the main separation of the samples along F1. EVOOs D1 and D3 were found to be similar to C4 and C5. EVOO D2 was comparable to A2 and opposite from D1 and D3. Sample D4 was positioned close to the origin. This result highlighted the better characterization of Abruzzo EVOOs and Liguria, Calabria, and Piemonte oils compared with the EVOO blend.

### 3.4. Discriminant Analysis

Linear discriminant analysis (LDA) was performed as a further unsupervised data elaboration, according to year, variety, and origin. It was applied to the first two principal component factors (F1 and F2), and results are depicted in confusion matrices (Table 5 and Tables S4 and S5, Supplementary Materials). The mean correct classification rates were 78.6% for vintage class (Table 5), 46.4% for geographical location (Table S4), and 32.1% (Table S5) for the cultivar class.

**Figure 4:**
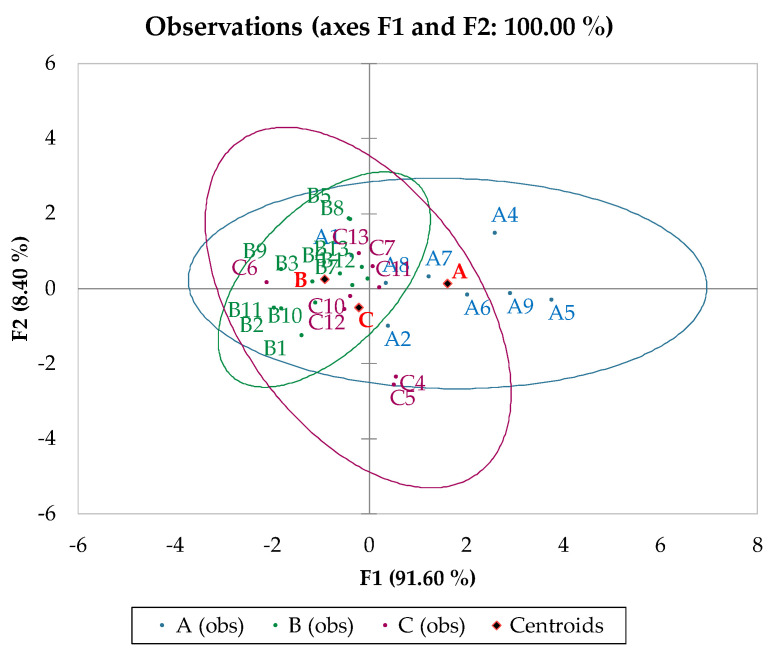
Observation plot of LDA.

The vintage discrimination supported the previous PCA evaluations, identifying the A samples’ distribution in the F1 direction, which were opposite to the compact B grouping, in the observation plot (Figure 4). Furthermore, samples C4 and C5 were spatially separated as evident C outliers along the F2 direction.

According to Table 5, the 2020 vintage samples were more accurately classified (91.7%) than those from 2019 and 2021 (75% and 62.5%, respectively). Indeed, from a graphical point of view (Figure 4), they were located closer to the reference centroid (B) compared with the A and C samples, where the distance between samples and their respective centroids was higher.

Concerning cultivar (Table S5), Don Carlo, FS17, Oliana, and Peranzana were correctly classified (100%), while Koroneiki, Lecciana, Leccino, and Dritta were completely misclassified (0%). Finally, the geographical locations correctly classified were Alanno, Casoli, and Loreto, while Pianella, Scerni and Vasto were misclassified (Table S4).

It is important to note that the low rate of LDA results related to the geographical location and the cultivar agree with the pairwise Kruskal–Wallis comparison outcomes, which did not identify significant differences among the EVOOs.

### 3.5. Cluster Analysis

**Figure 5:**
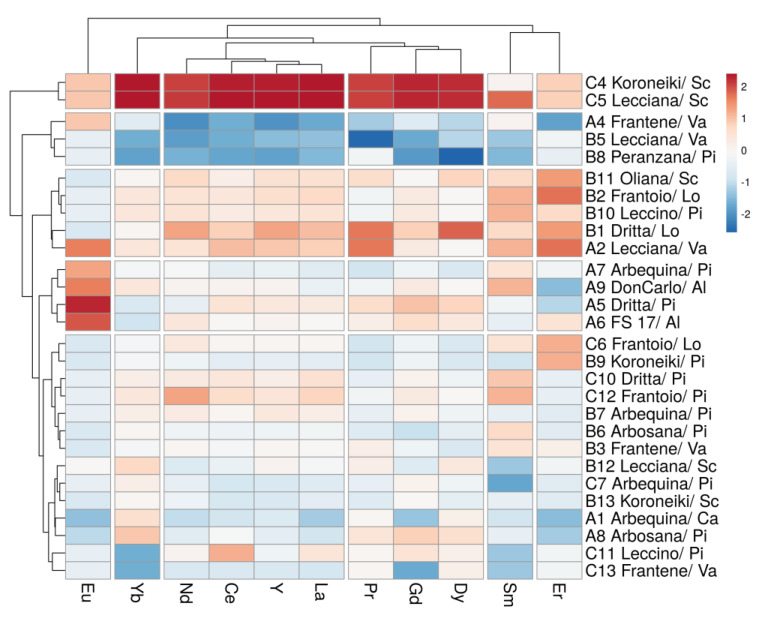
Heatmap plot of REEs and olive oils.

Aggregative hierarchical cluster analysis (HCA), using Euclidean distances and Ward’s linkage method, was implemented to interpret the chemometric data based on an input matrix consisting of 11 chemical variables (REEs) and 28 oil samples. The results of HCA are shown in a heatmap plot (Figure 5).

The column clustering highlights the correspondence of the graphical heatmap output information with a PCA loading plot (Figure S1). Eu, Er, Sm and Yb showed independent behavior and did not group with the two main clusters of La/Ce/Nd/Y and Gd/Dy/Pr.

The row clustering analysis identified four main groups, also highlighted in the score plots (Figure 3 and Figure 4), where C4 and C5 presented the highest concentrations of REEs, while EVOOs A4, B8, and B5, were characterized by the lowest concentrations of REEs. Moreover, EVOOs A5, A6, A7, and A9 presented the highest concentrations of Eu, while B1, B2, B10, B11, and A2 had the highest concentrations of Er.

HCA also confirmed that only the vintage influenced the REE concentrations’ tendency to form groupings, while geographical location and cultivar did not, as previously highlighted by the corresponding confusion matrices. Since the average annual temperature and cumulative precipitation were similar in the three considered years (data not shown), climate discrimination should be studied considering intrayearly (monthly and seasonal) climate trends, which could influence the specific phenological stages of olive tree development. Edaphic factors, such as the bioavailability of inorganic elements in soil and its chemical characteristics (pH, electrical conductivity, organic matter and inorganic carbon (CaCO_3_), should also be considered [71].

## 4. Conclusions

The overall results of this preliminary study show that in the high-EVOO-producing region of Abruzzo, the REE concentration patterns of olive oil, among different varieties, origins, and vintages, were almost homogeneous, showing the potential to be used as a marker of geographical origin. Among these three factors, only vintage slightly influenced REE concentrations, suggesting the possible effect of interactions among soil geochemistry, edaphic, and climatic characteristics. Some REEs are more effective and useful than others in representing the transfer of soil geochemistry to olive oil, in particular, Y, La, Ce, Nd, because of their larger contribution to the overall variance, having the strongest correlations, and the most similar patterns. The research outcomes add useful data to the scarce literature on the REE concentrations in olive oils, which are important for assessing food quality, especially in Mediterranean countries, where EVOO represents the main fat source, which is consumed daily. The use of the information gathered in this study can be a useful starting point for producing a reliable discrimination model that allows the verification and certification of the geographical origin of EVOOs produced in the Chieti and Pescara districts of Abruzzo Region. Future use of the developed procedure with larger data sets will verify its value. Furthermore, the relationships between EVOO samples originating from different areas and the effects of biogeochemical drivers on the geographical distribution of the elements require further exploration.

## Figures and Tables

**Table 1:** Olive oil samples: geographical location, sample coding, and cultivars.

Abruzzo Province	Geographical Location (Number of Samples)	Sample Code	Cultivar
Pescara (*n* = 18)	Loreto Aprutino (*n* = 2)	B2, C6	Frantoio
	Pianella (*n* = 2)	B10, C11	Leccino
	Pianella (*n* = 2)	A7, C7	Arbequina
	Pianella (*n* = 1)	A8	Arbosana
	Pianella (*n* = 1)	A3	Koroneiki
	Pianella (*n* = 2)	A5, C10	Dritta
	Alanno (*n* = 1)	A6	FS17
	Alanno (*n* = 1)	A9	Don Carlo
	Loreto Aprutino (*n* = 1)	B1	Dritta
	Pianella (*n* = 1)	B7	Arbequina
	Pianella (*n* = 1)	B8	Peranzana
	Pianella (*n* = 1)	B9	Koroneiki
	Pianella (*n* = 1)	B6	Arbosana
	Pianella (*n* = 1)	C12	Frantoio
Chieti (*n* = 11)	Vasto (*n* = 3)	A4, B3, C13	Frantene
	Vasto (*n* = 2)	A2, B5	Lecciana
	Scerni (*n* = 2)	B12, C5	Lecciana
	Scerni (*n* = 2)	B13, C4	Koroneiki
	Casoli (*n* = 1)	A1	Arbequina
	Scerni (*n* = 1)	B11	Oliana

Prefixes “A”, “B”, and “C” in the sample code correspond to 2019, 2020, and 2021, respectively. Numbers of samples are reported in brackets (*n*).

**Table 2:** REE concentrations (ng g^−1^) in olive oil samples. Mean and standard deviation (sd) of *n* = 3 replicates.

	Light							Heavy			
Sample Code	La	Ce	Pr	Nd	Sm	Eu	Gd	Y	Dy	Er	Yb
A1	1.28	2.93	0.37	1.19	0.27	0.09	0.27	2.01	0.37	0.18	0.37
A2	1.71	3.69	0.45	1.44	0.36	0.18	0.36	2.43	0.36	0.36	0.36
A4	1.18	2.59	0.31	1.02	0.31	0.16	0.31	1.65	0.31	0.16	0.31
A5	1.59	3.47	0.40	1.29	0.30	0.20	0.40	2.28	0.40	0.20	0.30
A6	1.52	3.24	0.38	1.43	0.29	0.19	0.38	2.19	0.38	0.29	0.29
A7	1.42	3.09	0.33	1.34	0.33	0.17	0.33	2.09	0.33	0.25	0.33
A8	1.36	3.22	0.39	1.27	0.29	0.10	0.39	2.05	0.39	0.19	0.39
A9	1.46	3.28	0.36	1.37	0.36	0.18	0.36	2.19	0.36	0.18	0.36
B1	1.75	3.60	0.45	1.58	0.34	0.11	0.39	2.53	0.45	0.34	0.34
B2	1.68	3.42	0.36	1.44	0.36	0.12	0.36	2.34	0.36	0.36	0.36
B3	1.52	3.21	0.38	1.36	0.33	0.11	0.33	2.18	0.33	0.27	0.33
B5	1.24	2.60	0.25	1.05	0.25	0.12	0.25	1.80	0.31	0.25	0.25
B6	1.49	3.15	0.34	1.32	0.34	0.11	0.29	2.12	0.34	0.23	0.34
B7	1.57	3.26	0.35	1.40	0.29	0.12	0.35	2.27	0.35	0.23	0.35
B8	1.22	2.55	0.36	1.09	0.24	0.12	0.24	1.70	0.24	0.24	0.24
B9	1.43	3.07	0.33	1.32	0.27	0.11	0.33	2.08	0.33	0.33	0.33
B10	1.63	3.37	0.36	1.44	0.36	0.12	0.36	2.29	0.36	0.30	0.36
B11	1.65	3.35	0.40	1.48	0.34	0.11	0.34	2.33	0.40	0.34	0.34
B12	1.50	3.13	0.38	1.25	0.25	0.13	0.31	2.19	0.38	0.25	0.38
B13	1.43	2.97	0.34	1.31	0.29	0.11	0.34	2.00	0.34	0.23	0.34
C4	2.02	4.20	0.47	1.71	0.31	0.16	0.47	2.80	0.47	0.31	0.47
C5	2.03	4.22	0.47	1.72	0.39	0.16	0.47	2.82	0.47	0.31	0.47
C6	1.53	3.27	0.33	1.42	0.33	0.11	0.33	2.18	0.33	0.33	0.33
C7	1.41	2.94	0.35	1.29	0.23	0.12	0.35	2.00	0.35	0.23	0.35
C10	1.65	3.42	0.35	1.41	0.35	0.12	0.35	2.24	0.35	0.24	0.35
C11	1.62	3.75	0.37	1.37	0.25	0.12	0.37	2.12	0.37	0.25	0.25
C12	1.70	3.52	0.36	1.58	0.36	0.12	0.36	2.30	0.36	0.24	0.36
C13	1.37	2.98	0.37	1.24	0.25	0.12	0.25	1.99	0.37	0.25	0.25
Mean	1.53	3.27	0.37	1.36	0.31	0.13	0.35	2.18	0.36	0.26	0.34
sd	0.21	0.40	0.05	0.17	0.05	0.03	0.05	0.27	0.05	0.06	0.05

**Table 3:** Pearson correlation coefficients.

Variable	Y	La	Ce	Pr	Nd	Sm	Eu	Gd	Dy	Er	Yb
Y	1.00										
La	0.97	1.00									
Ce	0.94	0.96	1.00								
Pr	0.79	0.75	0.78	1.00							
Nd	0.94	0.95	0.91	0.71	1.00						
Sm	0.59	0.58	0.55	0.36	0.62	1.00					
Eu	0.23	0.22	0.27	0.27	0.14	0.23	1.00				
Gd	0.81	0.81	0.85	0.69	0.80	0.48	0.38	1.00			
Dy	0.83	0.77	0.81	0.76	0.75	0.34	0.21	0.78	1.00		
Er	0.58	0.60	0.49	0.40	0.60	0.33	−0.04	0.29	0.29	1.00	
Yb	0.71	0.64	0.62	0.55	0.65	0.55	0.03	0.68	0.62	0.19	1.00

**Table 4:** Eigenvectors of PCA components F1 and F2.

	F1	F2
Y	0.365	−0.072
La	0.360	−0.092
Ce	0.358	0.021
Pr	0.307	0.102
Nd	0.353	−0.160
Sm	0.236	−0.028
Eu	0.103	0.761
Gd	0.328	0.242
Dy	0.316	0.127
Er	0.202	−0.544
Yb	0.275	−0.015

**Table 5:** Confusion matrix of samples grouped for vintage.

From\to	A	B	C	Total	% Correct
A	6	1	1	8	75.00%
B	0	11	1	12	91.67%
C	0	3	5	8	62.50%
Total	6	15	7	28	78.57%

## Data Availability

Data is contained within the article.

## Supplementary Materials

The following supporting information can be downloaded at: https://www.mdpi.com/article/10.3390/foods13010141/s1, Figure S1: PCA loading plot; Figure S2. Score plot of EVOO samples and foreign oils from Pisciottana, (Calabria region, D1), Frantoio (Piemonte region, D2), Taggiasca (Liguria region, D3), and EVOO blend (D4); Table S1. ICP-MS instrumentation and operating conditions; Table S2. LOD and LOQ values of elements in olive oils; Table S3. Range of REE concentrations in EVOO samples and those found in the literature (ng g-1); Table S4. Confusion matrix for the training samples based on geographical location; Table S5. Confusion matrix for the training samples based on cultivar.
