# Supplementary material for: Rare Earth Element Variability in Italian Extra Virgin Olive Oils from Abruzzo Region
Source: Foods. 2023 Dec 30;13(1):141. doi: 10.3390/foods13010141 (PMC10778968; doi:10.3390/foods13010141)
Supplement: Supplementary file 1 [file foods-13-00141-s001.zip › Supplementary Table.pdf]

SUPPLEMENTARY MATERIAL

Table S1. ICP-MS instrumentation and operating conditions

Instrument parameters	(Agilent 7900, Agilent Technologies, Tokyo, Japan)
Nebuliser	Nebulizer, MicroMist, U-series
Torch	Quartz glass torch
Spray chamber<br>Sample cone	Scott double-pass type at 2 °C<br>Nickel, 1.00 mm aperture
Skimmer cone	Nickel, 0.45 mm aperture
Plasma mode	General Purpose
RF power (W)	1550
RF matching (W)	1.80
Sampling depth	8 mm
Nebulizer gas	1.05 L/min
Auxiliary gas	1.0 L/min
Plasma gas	15 L/min
Sampling period (s)	0.31
Integration time (s)	0.1

Instrument parameters	(Agilent 7900, Agilent Technologies, Tokyo, Japan)
Repetitions	3
He flow	5.0 mL/min

Table S2. LOD and LOQ values of elements in olive oils

	LOD ng g^{-1}	LOQ ng g^{-1}
Sc	0.334	1.174
Y	0.104	0.147
La	0.107	0.210
Ce	0.197	0.373
Pr	0.026	0.051
Nd	0.079	0.140
Sm	0.048	0.112
Eu	0.005	0.011
Gd	0.031	0.075
Tb	0.003	0.007
Dy	0.021	0.044
Ho	0.004	0.009
Er	0.011	0.027
Tm	0.003	0.007
Yb	0.013	0.037
Lu	0.002	0.005
Th	0.053	0.106

Table S3. Range of REEs concentrations in EVOO samples and those found in the literature (ng g-1).

	Geographic area (method of analysis)		Y	La	Ce	Pr	Nd	Sm	Eu	Gd	Dy	Er	Yb
EVOOs	**Abruzzo**	Minimum	1.65	1.18	2.55	0.25	1.02	0.23	0.09	0.24	0.24	0.16	0.24
	**(ICP-MS)**	Maximum	2.82	2.03	4.22	0.53	1.76	0.47	0.20	0.47	0.50	0.36	0.50
		Range	1.17	0.86	1.67	0.28	0.74	0.23	0.11	0.23	0.25	0.20	0.25
		Median	2.18	1.52	3.27	0.36	1.36	0.32	0.12	0.35	0.36	0.25	0.34
		Mean	2.18	1.53	3.27	0.37	1.36	0.31	0.13	0.35	0.36	0.26	0.34
**Damak et al. (2021)**	**Tunisia**	Minimum		2.60	1.40	2.80			3.90		0.10		
	**(ICP-MS)**	Maximum		3.20	3.20	3.00			4.10		0.30		
		Range											
		Median											
		Mean											
**Astolfi et al (2021)**	**Italy**	Minimum		0.05	0.10	<0.008	<0.003				<0.005		
	**(ICP-MS)**	Maximum		0.79	3.50	1.65	13.80				0.055		
		Range		0.74	3.40	1.64	13.80				0.050		
		Median		0.11	0.20	0.009	0.04				0.005		
		Mean											
**Sayago et al. (2018)**	**Spain**	Minimum	0.22	0.50	0.93	0.09	2.28	0.06	0.01	0.06	0.04	0.02	0.02
	**(ICP-MS)**	Maximum	1.30	2.87	3.65	1.29	6.83	1.29	1.45	1.32	1.32	1.25	1.34
		Range	1.08	2.37	2.72	1.20		1.23	1.44	1.26	1.28	1.23	1.32
		Median											
		Mean											
**Aceto et al. (2019)**	**Italy**	Minimum											
	**(ICP-MS)**	Maximum											
		Range											
		Median											

		Mean		0.66	1.3	0.10	0.39	0.08	0.02	0.09	0.07	0.04	0.04
**Beltran et al. (2015)**	**Spain (ICP-MS)**	Minimum	0.20										
		Maximum	0.40										
		Range											
		Median											
		Mean	0.3										
**Farmaki et al. (2012)**	**Greece (ICP-MS)**	Minimum	0.20	0.24	0.42	0.06	0.10	0.14		0.03	0.10	0.06	0.02
		Maximum	1.15	5.31	6.46	0.52	1.67	0.45		0.35	0.30	0.24	0.25
		Range											
		Median											
		Mean	0.35	0.52	0.47	0.16	0.23	0.20		0.12	0.15	0.11	0.07
**Camin et al. (2010)**	**Italy (ICP-MS)**	Minimum		<0.002	<0.005		<0.004	<0.001	<0.0002				<0.0004
		Maximum		0.038	0.052		0.025	0.008	0.005				0.005
		Range											
		Median											
		Mean		0.013	0.028		0.008	0.003	0.002				0.002
**Benincasa et al. (2007)**	**Italy (ICP-MS)**	Minimum	0.08					0.004	0.004	0.004			
		Maximum	0.33					0.226	0.021	0.094			
		Range	0.25					0.222	0.017	0.090			
		Median											
		Mean	0.14					0.05	0.01	0.03			
**Poscic et al. (2019)**	**Croatia (ICP-MS)**	Minimum	<0.0005	0.001	0.001		0.001						
		Maximum	0.009	0.013	0.033		0.012						
		Range											
		Median											
		Mean											

Table S4. Confusion matrix for the training sample based on geographical location

from \ to	Alanno	Casoli	Loreto	Pianella	Scerni	Vasto	Total	% correct
**Alanno**	2	0	0	0	0	0	2	100.00%
**Casoli**	0	1	0	0	0	0	1	100.00%
**Loreto**	0	0	3	0	0	0	3	100.00%
**Pianella**	1	1	2	5	1	2	12	41.67%
**Scerni**	0	1	1	1	2	0	5	40.00%
**Vasto**	1	2	0	1	1	0	5	0.00%
**Total**	4	5	6	7	4	2	28	46.43%

Table S5. Confusion matrix for the training sample based on cultivar

from \ to	Arbequina	Arbosana	Don Carlo	Dritta	FS 17	Frantene	Frantoio	Koroneiki	Lecciana	Leccino	Oliana	Peranzana	Total	% correct
**Arbequina**	2	0	0	0	1	0	0	0	0	1	0	0	4	50,00%
**Arbosana**	1	1	0	0	0	0	0	0	0	0	0	0	2	50,00%
**Don Carlo**	0	0	1	0	0	0	0	0	0	0	0	0	1	100,00%
**Dritta**	0	0	1	0	0	0	0	0	0	1	1	0	3	0,00%
**FS 17**	0	0	0	0	1	0	0	0	0	0	0	0	1	100,00%
**Frantene**	0	0	1	0	0	1	1	0	0	0	0	0	3	33,33%
**Frantoio**	0	0	0	0	0	0	1	1	0	0	1	0	3	33,33%
**Koroneiki**	1	0	0	1	0	0	1	0	0	0	0	0	3	0,00%
**Lecciana**	0	1	0	2	0	0	0	0	0	0	0	1	4	0,00%
**Leccino**	0	1	0	0	0	0	1	0	0	0	0	0	2	0,00%
**Oliana**	0	0	0	0	0	0	0	0	0	0	1	0	1	100,00%
**Peranzana**	0	0	0	0	0	0	0	0	0	0	0	1	1	100,00%
**Total**	4	3	3	3	2	1	4	1	0	2	3	2	28	32,14%
